# Extracts from an Unpublished Report on the Medical Literature of Kentucky

**Published:** 1854-10

**Authors:** Lunsford P. Yandell


### Art. II.—EXTRACTS FROM AN UNPUBLISHED REPORT ON THE MEDICAL LITERATURE OF KENTUCKY.

BY LUNSFORD P. YANDELL, M. D., &c.

Dr. Daniel Drake, though a citizen of Ohio at the time of his death, spent a large portion of his active life in Kentucky, and is justly claimed by her as one of her Medical Authors. Having returned to his practice in Cincinnati, in 1818, at the breaking up of the Medical Faculty of Transylvania University of which he was a member, and having engaged in an unsuccessful effort to rear a Medical School in that city, he was induced to accept again the chair in Lexington, which he had resigned five years before. His first connection with this school gave rise to nothing of a public professional character that merits a notice in this Report, although previous to this time Dr. Drake had become an author of note. His "Picture of Cincinnati" had made him known to scientific men abroad, and he returned to Transylvania University with a high reputation for ability and learning. The public Lecture which he delivered introductory to his first course on Materia Medica, November, 1823, was published at the request of the Medical Class. The subject of the lecture was "The Incentives to professional study," and his manner of treating it was esteemed peculiarly happy. It was an eloquent discourse, abounding in thoughts calculated to excite the ambition and kindle the zeal of his pupils.

This inaugural address, we believe, is the only publication of which Dr. Drake's reunion with the Lexington Medical Faculty was productive. He resigned his professorship in Transylvania a second time, in 1827, at the close of his fourth course of lectures. In 1839 he was invited to Louisville, to a chair in the Medical Institute, just then entering upon its third session, and we find him in the winter of that year lecturing on Pathological Anatomy and Clinical medicine, and contributing again to the Medical literature of Kentucky. His Valedictory lecture to the graduating class was published. The subject of the lecture was the importance of clinical instruction to students of medicine. It was determined by the Faculty soon after Dr. DRAKE came into the school, to revive the medical journal which had been suspended after the issue of the second number, and he consented to become one of the editors. The first number of the *Western Journal of Medicine and Surgery* was published on the first of January, 1840. The name is nearly the same as that of the Journal which he had conducted in Cincinnati to the tenth volume, and of which he regarded this as a continuation. The pages of the first number were enlivened by some characteristic editorial articles from his pen, in which light, off-hand work, where the pun, the anecdote, wit, and humor are blended agreeably with science, he greatly excelled. During his connection with the journal he indulged freely his taste for such writing. His "Traveling Editorials," as he termed them, letters written to his colleagues during his tours to collect the materials of his great work since published, were read, perhaps, with as much interest as any other articles in the journal. He reported in the second volume (p. 343), a case of "Intermittent Fever, complicated with Hepatitis, Pneumonitis, Laryngitis, and Intestinal irritation." He closes his history of t h case with the following characteristic paragraph:

"To such of our readers as may think their time lost by the perusal of this article, we beg leave to say, that the afternoon (July 29), is oppressively hot; and that, if we were writing in as pleasant weather as they will read, we should no doubt forestall them in that criticism."

At the Medical Convention held at Frankfort, in January, 1841, he read an elaborate memoir on "the Disease called by the people 'Trembles,' and 'Sick stomach,' 'Milksickness,' as they occurred in the counties of Fayette, Madison, Clark, and Green, Ohio." He proceeds first to show by "way of exclusion," what agents cannot give rise to the disease, and arrives, by this process, at the conclusion that it is probably engendered by the *Rhus radican.* In his first object he is entirely successful; he proves most satisfactorily that milksickness cannot originate in any of the causes that have been heretofore assigned; but in the second he seems to us to have failed as signally as any who had written on.

the subject before him. His memoir, however, is a model of such investigations, and will be read with interest by those who are curious about the question of which it treats.*

The same Journal (vol. 6, p. 401), contains an interesting paper by Dr. Drake, the title of which is, "The Northern Lakes, a Summer resort for Invalids of the South." Its suggestions are judicious and important. It is written with the freshness of a traveler who had beheld the charming scenery he describes, and with the sagacity and judgment of an able, experienced physician.

In 1843 Dr. Drake engaged with a number of friends in the investigation of the phenomena of Mesmerism, which, at that time, was exciting great interest in Louisville. The point to which his attention was particularly directed was *clairvoyance*. In order to test the reality of this alleged faculty, he arranged that several gentlemen should write down a series of topics to be presented to the "subject" when in the mesmeric state, by reference to which, the answers being recorded, it would be seen whether the person mesmerised had before his mind the objects in the mind of the individual "in relation" with him. The test was fair, and at the same time decisive. The result was a conviction on the part of the majority of the committee, that there was no remarkable coincidence between the answers of the "subject" and the objects in the mind of the questioner. Dr. Drake published a detailed account of the inquiry under the head of "Analytical Report of a Series of Experiments in Mesmeric Somnambulism.† It will, perhaps, strike the reader as tedious, but it is conclusive of the question. The experiments proved that all was guess beyond what was suggested to the mind of the "subject" by the individual asking the questions. This Dr. Drake exhibited most clearly in a subsequent paper on the subject, entitled "Speculations on the Facts presented in the Analytical Report on Mesmeric Somnambulism." He shows in this article that a large proportion of the answers returned were not correct, that many were vague, and that those which seemed to favor the idea of clairvoyance were suggested by the questions proposed. We

*See Western Journal Med. and Surg., vol. iii.

†Western Jour. Med. and Surg. (New Series), vol i, p. 189.

have rarely read a paper displaying greater mental acumen than this, and we could not point to one which exhibits more of the peculiar traits of the author's mind. It will be found to contain the germ of the true theory of all those phenomena occurring in the conditions variously styled "clairvoyance," "biology," "hypnotism," &c. The explanation is, briefly, that the mind of the individual in the mesmeric state is in a peculiarly ductile condition, and may be directed, at will, by the person who may chance to have his ear when he passes into this state; or, as explained by Dr. Carpenter, he is a "thinking automaton," having no will of his own, but being completely under the guidance of the person in relation with him. Whatever the questioner sees, a word will cause the "subject" to see. But we need not go into a fuller explanation. We have said this much because at the time when Dr. Drake submitted his "Speculations" to the profession, the subject was very imperfectly understood, and he has the credit of having discovered the clue to phenomena which, to many highly cultivated minds, appeared mysterious and wonderful.

In 1850 appeared his great work on the Principal Diseases of the Interior Valley of North America,* a work which he had meditated for forty years. As long ago as 1823 he issued a printed circular to the physicians of the South-West, announcing his purpose to write a work on the diseases of the Ohio Valley, and soliciting the contribution of results afforded by their experience and observation. Engaged as he has been since in the labors of practice, and engrossed by the politics of medical schools, he has never lost sight of this noble enterprise. Amid all the changes through which he has passed in the Medical Colleges of three States, his plans in regard to this great labor of his life have remained unaltered. The institution founded by his exertions in early life in the city which he loved, the image of which has pursued him in all his subsequent journeyings, and made him restless and dissatisfied in every other institution with which he has been connected—an image beckoning him still to return to his

*A Systematic Treatise, Historical, Etiological and Practical, on the Principal Diseases of the Interior Valley of North America, as they appear in the Caucasian, African, Indian and Esquimaux varieties of its population.

early home, and disappointing him still as often as he yielded to its importunity--has never been able to divert his thoughts from this cherished object. It shows great steadiness of purpose that, amid every distracting care and throughout the unquiet life of the most active of medical politicians, the plan of his book was kept constantly before his mind. It is fortunate for his fame that these exciting concerns never wholly engrossed his thoughts.

About nine years before his work appeared he set earnestly about collecting materials for its composition, by exploring personally the great region of which it was to treat. Connected at the time with the Medical Institute of Louisville, and not disposed any longer to engage actively in the practice of physic, he enjoyed in the long vacations of the school the leisure necessary to the extended journeys required by the arduous enterprise. In the spring of 1841 he commenced a series of excursions in which he visited a wide area of country on the Mississippi, Ohio, Alabama, Tennessee, Missouri and St. Lawrence rivers, from the Balize to the Indian territory in Missouri, and from Pensacola Bay to Lake Superior. In the course of his travels he sought out the medical men everywhere, and collected their observations respecting the diseases of their several countries. It was his purpose to see the Great Valley in all its aspects, and its population in all its varieties and grades of civilization. He saw men in Barracks and in Indian wigwams, and made observations on the Caucasian, the African, the Indian, and the Esquimaux. And in addition to all this, he has availed himself of the observations of other travelers in remote parts of the Valley not explored by himself, thus embracing in his treatise the medical topography of the country from Vera Cruz to the regions around Hudson Bay.

It was to produce a work on the diseases of the Interior Valley out of materials collected in the region where they prevailed, that Dr. DRAKE was making preparation through this long period and by this extended travel. He never thought of framing a treatise out of other books. He meant that his work should be indigenous. No blind advocate of sectional medicine, he had yet observed enough to convince him that diseases were modified, to some extent, by the countries in which they prevailed. "That many physicians," says he, "overrate the degree of variation from a common standard which the diseases of different countries present, I am quite convinced; but feel equally assured that if the maladies of each country are studied and described without reference to those of any other, it would be found, if the state of medical science were equal in them, that the works thus produced would not be commutable, but that each would be better adapted, as a book of etiology, diagnosis and practice, to the profession and people among which it was written, than to any other."* It was his design to ascertain how far the diseases of the interior Valley differed from the same affections as described by writers elsewhere, and to make his work faithfully reflect these diversities.

The causes that go to modify disease are soil, climate, and population. Dr. Drake discusses these subjects at great length, four-fifths of his great volume being occupied with their consideration. Many have expressed regret that he should have given such prominence to etiology in a work designed for practical men; but to have given to it less space, or to have detached it from his treatise on the diseases of the West, as many would prefer that he should have done, would have been to abandon one of his most cherished ideas. He believed that the range of professional studies in our country was too limited, and it was his purpose to show, in this work, the full bearing of etiology upon practical medicine. He has presented the most extended system of medical topography to be found in any work extant. In accumulating and digesting this huge mass of details he was well aware, that, for the majority of medical men, he was performing a profitless and thankless labor. He foresaw that by the many his design would not be understood. But it was his theory of medical science, and no fear that his book would be less popular interfered with his putting it into practice.

As among the elements of Etiology, he begins with the mineral geology of the various regions described, holding that this is the proper basis of medical topography. It is well understood that the character of surface influences materially the health of a country, but no writer has indicated so clearly the relations of geological to medical science as Dr. Drake has done in this treatise.

He devotes two hundred pages of his work to the climtae of this

* Introd. Interior Valley.

great intramontane plane. And finally he presents a comprehensive view of its hygiene, or physiological and social etiology. He treats the subject not in a general way, but with special reference to our own population, the facts and conclusions having been derived from the author's own observation. As the climate of every country possesses something peculiar, so there are peculiarities in the hygienic condition of every people; and in one element at least the population of the great Valley stands more conspicuously than any other people upon earth; for where else, as Dr. Drake has pointed out, was there ever before such a commingling of races? Four out of five of the grand varieties of the human family are here—the Caucasian, African, Indian and Mongolian; and we have every branch of that family which stands first among the races—English, Irish, French, Italian, German, Scotch, Spaniard, Pole, Norwegian, Swiss, Swede, Hollander, Welch, and Jew, united here to form one great Caucasian family. For a century they have been mingling with each other and with the aborigines of the country, and the process of amalgamation which has been going on among men from the earliest ages, as shown by Dr. Drake, must go on in an accelerated ratio and here attain its final consummation.

"The great Central Valley of North America is the best crucible into which living materials, in great and diversified streams, can be poured for amalgamation. The double range of mountains which separate it from the Pacific Ocean, leave too little space for empire on that sea; and the detached communities which may there grow up, will be but deviations from the homogeneous millions, with which time will people the great region of the Appalachian and Rocky mountains, which is thus destined to present the last and greatest development of society."

Such are the broad, philosophical views in which this treatise of Dr. Drake abounds. This work is no compilation; it is no mere technical treatise on disease, but a scientific account of the characteristics of the great West, geological, ethnological, sanitary and climatorial.

The gifted author had the second volume of his treatise nearly ready for the press when summoned away from the scene of his earthly labors. His last published work was a small volume consisting of two Discourses, delivered before the Cincinnati Medical

Library Association, in January 1852. In the first of these he gives a biographical sketch of the early physicians of Cincinnati, with some incidents in his own eventful life; the subject of the second discourse is "Medical Journals and Libraries," of the rise of which he presents an interesting history.

The style of the first is light and sketchy, adapted to the simple annals which it was the purpose of the discourse to record. The second discourse abounds in fine reflections. In the following remarks, so full of the true spirit of philosophy and glowing with poetical beauty, the author might be supposed to be giving a description of himself:

"The enlightened aspirant for medical distinction is but little taken with the applause of the present moment, unless he perceives that after it has passed away there will remain some enduring element of fame—some solid and undecaying trunk, with fruit-bearing branches, which might have been overspread and hidden by a gorgeous drapery of leaves and flowers that enraptured the gazing populace for a day, but had no charms for him. When we feel in our hearts this indifference to the fleeting, and this warm regard for the permanent, let us believe that God has implanted the instinct for a wise and good purpose; and then follow it as a heavenly guide."

And again—in speaking of that class of medical works to which his own treatise confessedly belongs, he says:

"The true and healthful bibliothecal element of the mind consists of monographs, reports of original observations, and the systematic works which, in different ages, have presented the existing principles or acts of the profession, as they appeared to men of genius and experience. Such works have been at all times appearing and disappearing. They are not, like ordinary compilations, superseded by others of a later imprint, but a new generation overlooks them. They are like great men who have retired from the public gaze. They lie embalmed in the truths with which they are penetrated, and cannot decay; but like the bodies of kings and philosophers, steeped in spices and deposited in catacombs, they are found only in the unfrequented closets and alcoves."

No one, we think, who will look patiently through the writings of Dr. Drake, will fail to come to the conclusion, that they are penetrated by truths which will preserve them from decay, and cause them to be referred to by the medical philosopher and historian of future times. Our belief is, that few medical writers in our country have contributed to the profession a greater number of original observations, with a smaller admixture of error. In a diligent search through all his numerous publications it would not be easy to find one hasty, much less absurd, opinion, a single groundless hypothesis, or an idle speculation. He was a cautious writer. He was slow to avow his opinions. In nothing was he ultra. As a reviewer and critic he failed, because he was afraid to use the critical power which he possessed. He seemed reluctant to condemn or contradict. He speculated but little, and was never so much in his proper element as when putting forth his own observations. He loved research and phenomena. He was satisfied with nothing but facts. His mind was acute, analyitical, and logical, and if he had given himself up to it, he might have made one of the keenest of critics. It was a mind for observation and generalization, and he kept constantly up with the facts of the science, at the same time that no one surpassed him in the readiness with which he grasped and applied general principles. To sum up all in one word—*Dr. Drake was a philosopher*. He embraced in his capacious mind the whole ample range of medical science. He was eminently safe as a teacher, because he was cautious, candid, laborious and truthful. His style is uniformly simple, easy, and natural, sometimes loose and careless, but occasionally, when his subject favored it, ornate, poetical, and elevated. He had an active fancy, and his powers of description were of a high order, some of his pictures being models in their way. No one who has read it can ever forget his description of a "*doctor's shop*" as he was wont to contemplate it in the days of his pupilage, and as he still occasionally encountered it in his journeys through the country. The following description of the scenery of the Lakes is full of poetical beauty. He is describing what is seen from the ruins of Fort Holmes on the Island of Mackinac, and says:

"When the observer directs his eye upon the water more than upon the land, and the day is fair, with moderate wind, he finds the surface as variable in its tints as if clothed in a robe of changeable silk. Green and blue are the governing hues, but they flew into each other with such facility and frequency, that while still contemplating a particular spot, it seems, as if by magic, transformed into another; but these mid-day beauties vanish before those of the setting sun, when the boundless horizon of lake and land seems girt round with a fiery zone of clouds, the brilliant drapery of the skies paints itself upon the surface of the waters. Brief as they are beautiful, these evening glories, like spirits of the air, quickly pass away; and the gray mantle of night warns the beholder to depart for the village, while he may yet make his way along a narrow and rocky path, beset with tufts of prickly juniper. Having refreshed himself for an hour, he may stroll out upon the beach to listen to the serenade of the waters. Wave after wave will break at his feet, over the white pebbles, and return as limpid as it came. Up the straits he will see the evening star dancing on the ruffled surface, and the loose sails of the lagging schooner flapping in the fitful breeze; while the milky-way—Death's Path of the red man, will dimly appear on the waters before him. Behind, in the street, a lively group of Canadian French of every shade of color between white and red, will gossip and shrug their shoulders; on one side, should the Indians, who still inhabit the shores of lake Michigan, be on a visit to the Island, he will hear the uproar of a lodge of drunken Chippeways, with the screams of women and children, and the cackling of frightened hens; on the other, will see the sober and listless Ottawa, sitting in silent vacancy of thought, on his upturned birch canoe; his wife within the tent spreading cypress bark and flag mats upon the ground, as lodgings for the night; while half a dozen children loll or play about the door, and as many half-starved dogs curl up among them. Surrounded by such scenes, the traveler begins to realize that he is a stranger; when suddenly a new phenomenon appears and fixes the conviction. Every object becomes more visible; and, raising his eyes, he beholds the heavens illuminated with an aurora borealis, where he reads in fantastic characters of strange and eccentric light, that he is, indeed, a sojourner in a strange land, and has wandered far from home and his friends in the sunny regions of the South."

Dr. Drake, as is too well known to the members of this society, died in Cincinnati, on the 6th of last November, having returned to the city of his choice, and to the school which he founded thirty-three years before. His great work was left incomplete, but in such a state that the second volume will soon be ready for the press. The presence of this eminent man at our last meeting will be remembered with interest by many who were present on that interesting occasion. We saw him there as he appeared a quarter of a century before—still vigorous, active, buoyant, full of enterprise and hope—a pleasing instance of faculties unimpaired at an advanced age.

### JOHN E. COOKE, M. D.

It is natural to speak of Dr. Cooke in connection with Dr. Drake. They were for several years associated in the Medical Institute of Louisville. Both were at different times incumbents of the same chair in the Lexington and Louisville Medical schools. Dr. Cooke succeeded to the professorship of Theory and Practice of Medicine in Transylvania University when Dr. Drake resigned, in 1827; and when, in 1844, Dr. Cooke retired from that chair in the Medical Institute of Louisville, Dr. Drake was his successor.

Dr. John Esten Cooke was one of the most voluminous of the medical writers of Kentucky, having devoted several years of his life to systematic authorship. When he removed to Lexington, in 1827, he was engaged in the preparation of an elaborate work on Pathology and Practice,* one volume of which had already been published. The second volume was issued from the press at Lexington after he had entered upon his duties in the University. Before the appearance of this treatise, Dr. Cooke had made himself favorably known to the profession by one of the clearest and most philosophical essays on fever ever published.

The theory which he put forth in his treatise and repeats in several of his papers, is exceedingly simple, and has found in his pupils a host of advocates.

The first proposition he lays down is, that the remote causes of fever weaken the action of the heart, and that the principal of these are misamata and cold. The blood is blackened by the same causes, the quantity in the arteries is diminished, and an accumulation takes place in the vena cava or its great branches, in consequence of the weakened action of the heart. The effects of this accumulation are vertigo, headache, stupor and convulsions deranged secretion of the liver, kidneys, &c. The accumulation at length giving rise to convulsive agitation of the body, as in ague, the blood is urged vehemently on to the heart, which is excited to increased action by the increased supply of blood, and the hot

* A Treatise of Pathology and Therapeutics, 2 vols., pp. 800.

stage is developed. The difference between remittent and intermittent fevers arises out of difference in the excitability of the heart, which is so much greater in the former that the blood is not allowed to accumulate in such quantity as to produce an ague, as in the latter. All autumnal fevers are but modifications of one disease, and typhus, typhoid and yellow fevers, plagues, cholera, and dysentery are the same. Dyspepsia, liver complaints, and many of the diseases peculiar to females, depend upon congestion of the vena cava.

This is an outline of his pathology. His therapeutics are as simple—the great indication is to remove the congestion of the large internal veins; and this is to be effected mainly by cathartics. Occasionally venesection is beneficial; emetics are sometimes useful as evacuants; cold water is very effectual in reducing the action of the heart, and nauseants are also admissible with the same view; but the main reliance is upon evacuating the liver. Calomel stands incomparably first as a chollgogue, and is *the* remedy. It is not a matter of indifference what purgatives are given to aid its action—all are to be avoided that induce watery discharges. The passages must be consistent—bilious—and, after calomel, the articles most reliable for bringing them about are aloes, rhubarb, jalap, and scammony. The calomel is to be given in large doses and repeated until the evacuations become natural or the patient is well. In bad cases, Dr. Cooke says: "I have given with the best effect a drachm of calomel. It produces a copious, tenacious or viscid discharge, of a deep green or nearly black color, and affords the most marked relief." All the morbid phenomena depending upon congestion, the fever, whether intermittent, remittent, or typhus, give way when the accumulation of blood in the cava is removed by bilious purging. Hence, bark, tonics, and quinine are unnecessary. Salivation is to be avoided, if possible.

This, we believe, is a true account of Dr. Cooke's theory of Fever, and sufficiently full, we think, to be intelligible. In all his papers published after the appearance of his "Pathology and Therapeutics," he presents this view more or less in detail, and gives his treatment of disease as based upon it. The Transylvania Journal of Medicine contains many of his papers, and the burden of most of them is this pathology and practice, in the soundness of which he had the most undoubting faith. His first article in that Journal contains the "Theory of Fever," of which we have presented an outline. His second essay* treats of cholera infantum—its pathology and treatment—which he refers to the same cause—that excites fever, and treats in a similar way. He combats with unanswerable arguments the notion that the complaint is caused by dentition, worms, summer fruits, or cold, or heat unaided. The cause is malaria, he shows by a multitude of instances. Calomel is the curative agent. "As soon as consistent passages are established, the copious thin discharges cease." "So striking is the quieting effect of a dose of calomel, that parents have often inquired if there was not opium in the powder. The medicine must be continued daily until the passages become entirely natural in color, consistence, and smell. The diet may be anything that the child desires and can retain."

In the same volume of this journal he has an essay on Autumnal Diseases, in which he goes elaborately into the remote cause, adducing an irrefragable mass of facts in proof of their miasmatic origin. It is one of the most logical efforts to be found on that subject. If Dr. Cooke has not established the position, no future writer need hope for success. He has cited facts from every medical writer who had touched on the subject, and presents them with singular clearness and force.

This essay is followed in the same journal, by one on "Winter Epidemics," which he traces to the same remote cause. Persons in a neighborhood where miasmata abound are still under their influence, many on the verge of an attack of fever at the access of cold weather. When winter sets in there is another remote cause of fever in operation, cold, which produces a continuance of the epidemic through the winter months.† Pneumonia and pleurisy are only additional symptoms, intervening in many cases, the real disease being bilious fever. For the ordinary cases, the remedies are the same precisely with those proper in the treatment of autumnal fever. Where there are pneumonic symptoms, it is necessary in addition to resort to bleeding and blistering.‡

---

* Trans. Jour. Med., vol. 1, p. 59. Ibid. p. 190.

† Trans. Jour. Med. &c., vol. 1, p. 539. ‡ *Ibid* p. 550.

The second volume of this journal contains an essay by him on the "Remote cause of Typhus Fever, with some observations respecting its treatment." He opposes its contagiousness, but insists that it rises like the other fevers, from the decomposition of vegetable matter. "Typhus fever, therefore," he says, "is common autumnal fever or bilious remittent fever of England,—p. 85. Purgatives are the most useful remedies. He would not urge his patients to eat.

In this Dr. Cooke is opposed to the more matured experience of the present day, which condemns purgatives, and inculcates the administration of food at regular periods, whether the patient has appetite or not.

In the same volume we find from him an essay on Phlegmasia dolens; and one on the use of purgatives in the diseases of women. Phlegmasia dolens he treats by cathartics, as he does menorrhagia, uterine hemorrhage, &c., which he holds, are produced by that prevalent venous congestion which is, to his mind, the source of so much trouble.

The fourth volume of this journal contains an able and acute paper by him in which he examines Clutterbuck's and Broussai's theories of fever. He succeeds in demonstrating very clearly that one erred as much as the other in his location of a disease which has no topical origin. He was eminently successful in demolishing the structure which these gifted men had reared with so much ingenuity; but the theory which he would have substituted for theirs has gone into the same "limbo of vanity"—that *gurgite vasto* where so many theories of fever have been swallowed up and lost.

In the same volume he has a long, candid, and manly reply to some strictures on his Pathology and Therapeutics in the American Journal of the Medical Sciences.

The 5th volume contains two of his papers—the first, on the use of cold water in fever—an admirable paper, founded on experience, and inculcating a salutary practice; the second, on Spasmodic Cholera, of which at that time (1831) he had not yet seen anything.

The succeeding volume contains two papers from his pen. The subject of the first, is some of the diseases incident to a state of pregnancy. He recommends the use of purgative medicines as mitigating the sufferings—the headache, nausea, vomiting, &c.—of pregnant women—a practice suggested by his theory of congestion, but which, there is reason to believe, will often be found to produce happy consequences.

His second paper consists of "Remarks on Cholera as it appeared in Lexington, in June, 1833."

This is a remarkable paper. It records the observations made by him during the progress of the pestilence in Lexington. He describes with singular clearness the topography of the town and the spread of the epidemic, and gives with faithful minuteness an account of his extraordinary practice. He labors hard to prove that the disease conforms to the laws that govern malarious diseases, and it must be admitted makes out a strong case. He shows that it broke out in just such weather as is most favorable to the generation of malignant fevers, and that it was most fatal in precisely those localities where such fevers are most apt to prevail. His argument is as strong as any ever produced in favor of the miasmatic origin of cholera.

But the most striking feature in this article is his treatment. He administered calomel to his patients laboring under cholera with a boldness that, perhaps, has never been equalled. In numerous cases he gave an ounce, and even a table spoonful, at a dose. One young man, William Douglass, 19 years of age, took a table spoonful every six hours for three days. Mercurial purging was never effected, and the patient died. But another patient recovered under similar treatment, and still lives to bear witness to the amount of calomel that a man may take without injury to his system. Mr. Britton took a table spoonful four times on one day, and three times the next, "and in the same time three other similar doses, having thrown up three." The doses were repeated immediately after the regular ones were rejected. Dr. Cooke adds, "he was somewhat salivated," but his final recovery was complete.

In recording this extraordinary practice we feel that we owe it to justice to testify, as we are able to do from personal observation, that Dr. Cooke was as successful in his practice as any of his brethren in Lexington.

Connected as Dr. Cooke was with a leading medical school, having a manner which at once convinced every hearer of the sincerity of his opinions and the truth of his statements, with great logical powers and a style of remarkable clearness, it is not surprising that through his writings and lectures his theory gained a wide currency, and his practice was extensively adopted. But a quarter of a century has passed away since his system was promulged, and we need not add that it has not escaped the fate that has attended every other system of medicine hitherto proposed. The theory is no longer defended by any of his pupils, and among them all, embracing many of the most eminent and skilful practitioners of the West, very few, perhaps, would venture upon his heroic practice. But let us be just to this honest and laborious cultivator of his profession. If his theory of disease has not stood the test of the severe scrutiny which awaits all medical speculation, his facts will remain, and his histories of cases form a record which will not lose its interest in all coming time. No one who reads these histories will doubt the perfect candor in which they are written, or fail to be struck with the lucid and simple style of the narrative. Honesty of purpose marks everything which has emanated from the pen of Dr. Cooke. It is visible in every page of his voluminous writings. He sought truth and truth only. He never contended for victory, but for principle. No man ever entertained fuller convictions of the truth of all that he inculcated. He was, happily, without a doubt of the soundness of his pathology and of the entire efficiency of his practice. His works are models of a clear, direct, simple style. It was his study to communicate his thoughts in the fewest and simplest words, and in a manner the most remote from circumlocution. He is as free from affectation as it is possible to be, and nearly as destitute of any semblance of art. One would look in vain through all his papers on whatever subject for a single specimen of an ambitious or fine style. He attempted no ornament, and seems never to have thought of rounding off a period. His sentences flow along smoothly, and pleasingly—as naturally as he would have spoken them around his own fire-side. It has been remarked, that few men can be trusted to write their own biographies. Dr. Cooke is one of the few who, without any detriment to his fame, might have performed that delicate work. He would have written his life with all the honesty that he lived it—with perfect impartiality, keeping nothing back through a weak vanity, and exaggerating nothing.

Dr. Cooke died, at his farm, in Trimble county, the day on which this report was read, (Oct. 20th, 1852.)

---

### DR. JAMES BLYTHE.

---

Each medical professor in Transylvania University feeling called upon to aid in the sustentation of the Journal of the school, James Blythe, D. D., Professor of Chemistry, wrote two papers for the initiatory numbers. Dr. Blythe was a Presbyterian clergyman of a strong and cultivated mind, and was one of the early presidents of Transylvania University. He was of a decided and even stern orthodox faith, having been reared in the same neighborhood, in N. C., with his colleague, Prof. Caldwell, to whom as an older and more advanced scholar, he was in the habit of relating, he was indebted when a lad for assistance in mastering many a hard lesson. He seems to have entertained serious suspicions of the orthodoxy of his early school-fellow, and to have felt himself called upon to keep a sharp watch over his prelections. Certain it is that there was a perpetual controversy between these teachers as long as Dr. Blythe remained in the school. The *odium theologicum* helped to point the arguments of the physiologist against the chemist; and the chemist, in turn, was harder upon the doctrines of the physiologist because of the suspicion that they tended to infidelity.

In his first paper (1828) Dr. Blythe treats of the "*Chemical changes that take place on the atmospheric air and upon the blood during respiration.*" He gives the leading facts on the subject as they were then received, in a calm, scientific, argumentative manner, which contrasts strikingly with the manner of his colleague on the same question. He says: "The whole of animal and vegetable developments are by a set of philosophers consigned to the government of a power denominated Vitality. But we are not told by what law this power maintains its sway. Now this is the very thing we are in quest of, and we must still insist upon having those laws classed and explained. This vitality savours very much of the occult qualities of Aristotle." It is but too evident that he had here his physiological colleague in his eye, as he must have done when he penned the following passage in his second article, on respiration and animal heat:

"But suppose it should be said that none of these substances are found in the blood in a *free* or formal state. On this supposition I should like to know how we are to account for the two following experiments, viz: that the serum of blood changes the syrup of violets to a green, if blood does not contain *free* soda or some alkali; and if there be no free carbonic acid in the blood, how can we account for the fact, that newly drawn blood, under an exhausted receiver, gives it out in considerable quantities."*

We are not informed what answer the physiologist returned to these questions of his colleague, but we confess that they appear to us still unanswerable upon the hypothesis of the vitalists.

Dr. Blythe contends for Crawford's theory of animal heat, to produce which "he has no doubt, is one of the chief objects of respiration." Here again he was in antagonism to his ancient school-mate, of whom he speaks as "a gentleman of great philosophical pretensions," who claimed to have "caused Dr. Cooper† to abondon his ground" in support of the chemical theory of this process. Dr. Blythe thinks that Dr. Cooper was too ready to yield his ground, and we imagine the clear-sighted old philosopher came to think so himself before he died.

With these sensible papers, which havs not suffered at all by the subsequent progress of physiological science, Dr. Blythe's contributions to our medical literature seem to have ended, however much he continued to chafe his fiery colleague by assailing his peculiar notions as often as opportunity was afforded. He resigned his chair in the institution in 1831, and died, in 1841, in the 72d year of his age, having been for several years President of Hanover College, in Indiana.

---

* Transylvania Journal, vol. i, p. 333.

† Late President of South Carolina College.

### WILLIAM H. RICHARDSON, M. D.

The circumstance that drew Dr. Blythe for a short season away from more congenial studies to the public discussion of points in physiology brought out his colleague, Dr. Richardson, as an author. Dr. Richardson had been for several years a public teacher. As early as 1812 he gave lectures on midwifery to a few students in Lexington. In 1817 he was appointed professor of obstetrics and diseases of women and children, before he had taken the degree of M. D.; and on the organization of the medical faculty, in 1819, he was recalled to the same chair, which he continued to fill until he died. The second number of the Transylvania Journal contains a paper by him on "*Secale cornutum*," in the use of which his extended practice had afforded him much experience. He expresses the belief that the remedy, invaluable as it is, was too frequently resorted to, and that more confidence was reposed in it by accoucheurs "than comports with the legitimate objects of medical science."

In the second volume of this journal, p. 46, we find reported by him an interesting case of "Preternatural membrane in the vagina, requiring an operation during labor."

These brief papers give proof of the sound, judicious practitioner, but of one unaccustomed to making a public record of his experience. Dr. Richardson's use of language was not happy. He was not a scholar; but his oral delivery was fluent, easy, natural, earnest, and agreeable, and, without being a profound or scientific teacher, he was a popular medical instructor. He died, before his powers of mind or body had been impaired by age, of fever, on the 14th of September, 1845.

